# One way or another…or both: Different roles of fathers, mothers, and adolescents in the intergenerational transmission of inclusive attitudes

**DOI:** 10.1111/famp.13023

**Published:** 2024-06-24

**Authors:** Fabio Maratia, Elisabetta Crocetti

**Affiliations:** ^1^ Department of Psychology Alma Mater Studiorum University of Bologna Bologna Italy

**Keywords:** adolescents, AMIP, attitudes, families, integration, parents, transmission

## Abstract

This study aimed to examine the processes of intergenerational transmission of attitudes toward migrant integration policies in families with adolescents. Participants included 809 adolescents (*M*_age_ = 15.61, range: 13.87–20.04 years), 545 fathers (*M*_age_ = 51.19, range: 38–77 years), and 716 mothers (*M*_age_ = 48.11, range: 33–68 years) involved in a longitudinal study with two assessments and a time‐lag of 1 year between them. Each family member completed the Attitudes towards Migrant Integration Policies scale. In addition, adolescents reported their perception of discussion of current events with parents and the level of support they receive from them. The cross‐lagged model highlighted a unidirectional transmission, with fathers' but not mothers' attitudes toward migrant integration policies influencing adolescents' attitudes. Furthermore, it has been examined which factors can either amplify or reduce the strength of intergenerational transmission processes considering individual characteristics of both adolescents (i.e., sex, age) and their parents (i.e., political orientation), and cultural (i.e., family, ethnic background) and relational (i.e., discussion of current events, perceived support from fathers and mothers) family characteristics. Individual factors (i.e., fathers' political orientation) and family relational characteristics (i.e., perceived support from fathers) moderated the transmission processes. The transmission was bidirectional when fathers reported being left‐wing and politically oriented and stronger when adolescents reported high support from their fathers. Thus, this study underscores the complexity of the family context, highlighting that the transmission of inclusive attitudes does not always operate in one way (e.g., from parents to children) or another, but in some cases, simultaneously.

## INTRODUCTION

Nowadays, families navigate through social contexts characterized by increasing ethnic and cultural ddiversities, with the global migrant population reaching 281 million in 2020 (McAuliffe & Triandafyllidou, 2021). Such diversity poses complex challenges for families from both majority and minority groups. On the one hand, families from the majority group may perceive the changes resulting from multiculturalism as a threat, which could lead to more intergroup tensions instead of harmony (Kunst et al., 2021). On the other hand, families with a migrant background (i.e., families with adolescents born outside the destination country or with at least one parent born outside the destination country; European Commission, 2023) have to adjust to the host societies overcoming various barriers (e.g., educational, cultural, or economic; Medarić et al., 2022). Integration policies implemented by countries can help to manage these challenges by facilitating the adjustment of migrant families while at the same time fostering more social cohesion and well‐being, which can also benefit families from the majority group (Esser, 2004).

Various actors play a key role in the development of these policies. National integration policies proposed by governments are intertwined with public opinion, and their endorsement and implementation also depend on citizens' inclusive or nontolerant attitudes (Callens, 2015). Thus, it is essential to understand which factors can promote individuals' openness to these policies. In this vein, focusing on young people is fundamental to understanding how inclusive attitudes are formed in a critical formative phase of life, such as adolescence, where the development of positive values (Padilla‐Walker et al., 2018) and more inclusive attitudes (Albarello et al., 2020) is strongly affected by the contexts within which young people are embedded (Bronfenbrenner & Morris, 2007).

Family represents the first socialization context for adolescents, with parents acting as a point of reference, transmitting values and attitudes to their children over time (Crocetti et al., 2023; Degner & Dalege, 2013). Thus, this study aimed to identify which figure (e.g., fathers) can influence other family members (e.g., children) inclusiveness by transmitting their attitudes. Moreover, this study sought to investigate which characteristics of adolescents, parents, and families can be crucial to facilitating the intergenerational transmission processes.

### The integration of migrant families

The integration of people with a migrant background is becoming vital in today's diverse societies. The European Union in 2022 registered the presence of 1.9 million migrants from non‐EU countries (The Statistical Office of the European Union, 2022). In Italy, where the study was conducted, in the same year, the number of individuals with a migrant background was around six million (Istituto Nazionale di Statistica [ISTAT], 2023), making this country one of the major immigrant destinations in Europe (United Nations, 2019). Migrants come from various countries, mainly Eastern Europe (e.g., Romania, Albania, and Ukraine), North Africa (e.g., Morocco), and Asia (e.g., China). Most of them (56.3%) come to Italy for work reasons, with an additional 40.3% for family reasons, such as family reunions (ISTAT, 2023). Furthermore, Italy represents one of the European countries with the highest concentration of high school students (10%) with a migrant background (International Organization for Migration, 2020; Organization for Economic Cooperation and Development & European Commission, 2023). This increasing diversity is also observed in the regional context of this study, the Emilia‐Romagna region in the North‐East part of Italy, which records the highest presence of individuals with a migrant background both in the general population (12.8%; Regione Emilia‐Romagna, 2023) and in the school context (11.8%; Unione Italiana del Lavoro Scuola, 2023) compared to all other Italian regions. Given these numbers, managing such diversity becomes crucial and requires a better understanding of the best strategies to promote the positive integration of families with different cultural backgrounds into society.

A ‘successful’ integration process is ensured when migrant families experience positive outcomes in different life domains (Ager & Strang, 2008). These domains are related to four main themes: markers and means (i.e., access to employment, housing, education, and health), social connection (i.e., the creation of social bridges, bonds, and links), facilitators (i.e., overcoming linguistic and cultural barriers while experiencing safety and stability), and foundation (i.e., citizenship and rights). These areas are essential for a positive integration process, and each appears to be interconnected. For instance, promoting good housing conditions for migrants can simultaneously increase opportunities for social connection (Ager & Strang, 2008). One way to foster these positive outcomes is to promote at the country level the creation and implementation of new policies that encompass these key domains (European Commission, 2020). According to the Migrant Integration Policy Index (MIPEX; Solano & Huddleston, 2020), comprehensive integration is ensured when governments develop policies that ensure basic rights, equal opportunities, and a secure future for people with a migrant background.

The development and implementation of integration policies largely depend on the political agendas of the government of a given country. Political movements and, therefore, governments in Italy and most of Eastern and Western Europe are organized along the left–right‐wing distinction, indicating individuals' political attitudes and beliefs (Wojcik et al., 2021). While left‐wing parties generally remain open to the cultural, social, and political integration of individuals with a migrant background, right‐wing parties tend to emphasize immigration‐related issues (Massetti, 2015) and advocate for anti‐immigrant political agendas (Santi Amantini, 2022). However, in countries where integration policies are more developed, positive outcomes, such as higher individual inclusiveness (Gregurović, 2021) and overall greater social cohesion (Albarosa & Elsner, 2022), are achieved. If several instruments, like the MIPEX, have been proposed to monitor to what extent countries implement integration policies, less is known about how individuals perceive them. In this vein, it should be crucial to understand better how attitudes toward migrant integration policies are developed in the formative period of adolescence and if the family context positively or negatively influences these inclusive attitudes.

### Parents and children similarities: when is transmission?

Adolescence is a crucial period for the development of inclusive attitudes. In this period, youth become more autonomous and develop their own identities (Crocetti, 2018) while experiencing a wide range of diversity. The way they approach this diversity and, thus, the likelihood of developing more or less inclusive attitudes may depend on certain individual characteristics of adolescents (Svensson & Syed, 2023). In this regard, both sex (Maratia et al., 2023) and ethnic background (van Zalk et al., 2013) appear to influence adolescents' attitudes and orientation toward minority groups, suggesting that being female and having a migrant background are generally associated with more openness to diversity. Furthermore, as adolescents grow up and refine cognitive abilities and moral reasoning, they might show more empathic competencies (Bobba & Crocetti, 2022), consequently developing a more inclusive orientation toward diverse others (Bayram Özdemir et al., 2021). Nevertheless, together with individual characteristics, contexts, from the most proximal (e.g., family) to the more distal (e.g., culture) ones, can also influence adolescents' inclusiveness (Raabe & Beelmann, 2011).

Family is one of the primary socialization contexts for adolescents, with parents significantly influencing their development of attitudes, values, and beliefs. A vast corpus of literature based on cross‐sectional studies (for a review, see Degner & Dalege, 2013) has pointed substantial similarities in adolescents' and parents' values that are crucial for their adjustment to society, such as openness to change (Dubrov & Tatarko, 2018), prosocial orientations (Albert & Ferring, 2012), and collectivist values (Albert et al., 2009). Furthermore, parents and their children hold similar attitudes that help them navigate into their intergroup relationships, such as positive or negative attitudes toward migrants (Jaspers et al., 2008), political attitudes (e.g., party identification; Meeusen & Boonen, 2022), and ideological orientation (Rico & Jennings, 2016).

Although these studies consistently point to similarities in adolescents' and parents' values and attitudes, their cross‐sectional design does not allow for uncovering intergenerational transmission processes. Intergenerational transmission represents a series of processes through which one generation influences the next generation directly or indirectly (Vollebergh et al., 2001). On the one hand, parents can influence adolescents' development by acting as models (Wiese & Freund, 2011) and directly communicating their own values, attitudes, and beliefs (Acock & Bengtson, 1980). Indeed, according to the social learning theory (Bandura, 1977), children learn from their parents' attitudes or beliefs and create a view of society similar to those of their parents. On the other hand, growing up within a particular social context and inheriting their parents' social status may result in children indirectly internalizing values, norms, and beliefs prevalent within their family's social sphere (Glass et al., 1986; Min et al., 2012). Whether direct or indirect, the transmission of values and attitudes from parents to children represents processes that occur over time (Crocetti et al., 2023). Thus, a longitudinal approach is necessary to unravel this type of influence.

Results from studies with at least two‐time points have confirmed that parents can influence their children's cultural values (Perez‐Brena et al., 2015; Vollebergh et al., 2001), political orientations (e.g., right‐wing party preferences; Oepke, 2008), and their attitudes toward people with a migrant background (Gniewosz & Noack, 2015; Miklikowska et al., 2019) over time. However, it is not clear which is the potential role of each family member in these transmission processes. For instance, despite evidence suggesting that mothers typically spend more time with their children (Folbre et al., 2005), and thus have more opportunities to socialize and influence them (Jaspers et al., 2008), longitudinal studies show that parents have a similar impact on adolescents' attitudes and beliefs (e.g., Boonen, 2017; Gniewosz & Noack, 2015).

Furthermore, intergenerational transmission is not necessarily a unidirectional process in which parents have unilateral influences on their children but can also be conceived as an interactive process in which children can influence their parents (Acock & Bengtson, 1980; De Mol et al., 2013; Kuczynski et al., 2016). Thus, these processes could also be bidirectional, wherein adolescents have an active role in negotiating their new values and attitudes with those of their parents and vice versa (Akyil et al., 2016; Barni et al., 2023). In this regard, only a few studies have highlighted this interactive effect (e.g., Perez‐Brena et al., 2015), with the majority of the results indicating that the impact of parents on their children is more substantial than the other way around (Vollebergh et al., 2001), and this may be since parents hold more stable beliefs and attitudes than their children (Crocetti et al., 2016). In light of this, more longitudinal research is needed to disentangle reciprocal associations between parents and their children's attitudes toward migrant integration policies and to tackle which factors can moderate it over time.

### Promoters and hindrances of the intergenerational transmission processes

The intergenerational transmission processes are complex phenomena that can be enhanced or hampered by different individual and contextual factors (Meeusen & Boonen, 2022). In this vein, how this phenomenon unfolds over time can depend on each family member, considering both *adolescents'* (i.e., age and sex) and *parents'* (i.e., political orientation) individual characteristics. In addition, *family‐related* characteristics pertaining to their ethnic background and relationships (i.e., discussion of current affairs and perceived support from parents) can moderate the transmission processes of inclusive attitudes.

#### Family Members' characteristics

Adolescents' *sex* might play an important role in the parent–child influence. For instance, some evidence has shown that adolescents' gender role attitudes are more influenced by their same‐gender parents (Filler & Jennings, 2015). Furthermore, when examining political orientation, specifically regarding right‐wing preferences, congruence among adolescents and their parents was stronger in father‐son dyads than in father‐daughter dyads (Van Ditmars, 2023). In contrast, other studies demonstrated the absence of any gender‐match effect in the association between parents' and children's attitudes (Augustijn, 2022; Bucx et al., 2010; Degner & Dalege, 2013; Duriez et al., 2008; Meeusen & Dhont, 2015). Thus, there is no conclusive evidence yet about the moderating role of adolescents' sex.

Besides their sex, adolescents' *age* and developmental processes can also affect the transmission of values and beliefs from parents to adolescents. In this vein, school, specifically classmates, represent an important point of reference for adolescents' intergroup attitudes, helping them navigate the social world (Albarello et al., 2020; Thijs & Verkuyten, 2013). Thus, the more adolescents grow up, become autonomous, and interact with peers, the more parents' influence is expected to decrease and leave space for the influence of peers (Alfieri & Marta, 2012; De Goede et al., 2009). Some supporting evidence of this principle has been provided in a meta‐analysis on values related to the work sphere (Cemalcilar et al., 2018) according to which fathers‐child similarities of their work values (e.g., work ethics) decrease over time, while peers' influence gains more importance. However, longitudinal evidence that has simultaneously analyzed the impact of parents and classmates on adolescents' attitudes has found that despite the significant effect of peers, the influence of parents remains significant over time (Bobba, Branje, & Crocetti, 2024). Thus, while peers may gain importance over time, parents maintain their influence. In light of this, adolescents' age should be further considered to understand better if it moderates the transmission of positive attitudes toward integration policies.

In addition to adolescents, mothers' and fathers' characteristics can play a significant role in modulating the intergenerational transmission processes. Parents' *political orientation* may impact inclusive attitudes. How individuals see immigration has become an organizing principle of political attitudes (Rekker, 2016). At the same time, populist right‐wing parties with an anti‐immigrant agenda have increased (Santi Amantini, 2022). Thus, aligning with right‐wing parties may negatively influence families' inclusiveness, with evidence suggesting that individuals who support far‐right parties show negative attitudes toward integration policies (Howard, 2010). However, if parents' political orientation is likely to affect family members' inclusiveness directly, less is known about their potential moderating role in the transmission processes. Thus, how can this aspect reinforce the transmission of attitudes from parents to their children and vice‐versa? To answer this question, it is crucial to uncover further the factors that can moderate the intergenerational transmission of positive attitudes toward migrant integration policies.

#### Family‐related characteristics

Since family represents a complex system, adolescents' and parents' characteristics are not the only factors to consider to understand what facilitates the transmission processes. In this vein, the family's *ethnic background* and *relationships*, such as the discussion of current events and the perceived support from fathers and mothers, can play a crucial role in promoting the transmission of values and attitudes.

*Family ethnic background* might impact family members' views regarding different intergroup topics (for a review, see Hughes et al., 2006). Families from minority groups have been found to engage in frequent cultural, racial, and ethnic socialization practices. Through this transmission of information about their background, parents also aim to inform their children of potential challenges and social barriers they may face (Elias et al., 2022; Ream, 2023). These socialization practices could make adolescents with a migrant background more aware of the importance of integration policies to overcome these barriers. As a result, they may exhibit more positive attitudes toward these policies and, generally, a greater similarity with their parents than adolescents from majority groups.

Together with family cultural background, family relational factors play a crucial role in the transmission processes. Since the transmission of attitudes and values represents a social learning process (Kuczynski & Parkin, 2007), the likelihood of families engaging in *discussion* about topics such as intergroup relations could represent another important factor in facilitating the transmission of positive attitudes toward the integration policies. For instance, the more intergroup attitudes are openly discussed within the family context, the more parent and adolescent similarities increase (Degner & Dalege, 2013). Furthermore, when adolescents can discuss social issues and political topics with their parents, they are likely to show political attitudes (Meeusen & Boonen, 2022) and prejudice levels (Meeusen & Dhont, 2015) similar to those of the latter.

Finally, if engaging in discussion of these current events with parents is likely to facilitate the social learning process, it is of utmost importance to consider also the climate in which these discussions occur. In this regard, the *support* adolescents perceive from their parents can play a significant role. For instance, the greater the emotional proximity and perceived support between parents and their children, the more the family transmission of attitudes and values is facilitated (Augustijn, 2022; Brown et al., 2018; Miklikowska, 2016). In conclusion, considering all these factors, ranging from individual to family ones, could highlight which characteristics and processes may facilitate the transmission of positive attitudes toward integration policies and promote inclusivity in ethnically and culturally diverse societies.

### The present study

Given the importance of integration policies in promoting more general well‐being within societies, this study aimed to understand how attitudes toward these policies are developed in families with adolescents. In particular, we sought to disentangle how parents' and adolescents' attitudes toward migrant integration policies are intertwined over time. Given that parents are likely to have more stable attitudes than their children, we expected a predominant unidirectional effect, with mothers' and fathers' attitudes toward migrant integration policies predicting adolescents' attitudes more than the other way around.

Furthermore, we examined which factors can either amplify or reduce the strength of intergenerational transmission processes considering individual characteristics of both adolescents (i.e., sex, age) and their parents (i.e., political orientation) and cultural (i.e., family ethnic background) and relational (i.e., discussion of current events, perceived support from fathers and mothers) family characteristics. While we examined some of these factors (i.e., adolescents' sex and parents' political orientation) with an exploratory approach because of the inconclusive or limited evidence available in the literature, we formulated more specific hypotheses for others. Specifically, considering adolescents' age, we hypothesized that parents' influence will decrease when adolescents grow up. Concerning family‐related characteristics, we expected the transmission processes to be more robust in families with a migrant background. Moreover, we predicted that high levels of discussion of current events and fathers' and mothers' support would facilitate the intergenerational transmission processes of inclusive attitudes.

## METHOD

### Participants

Participants for this study were drawn from the ongoing of the longitudinal project IDENTITIES “Managing identities in diverse societies: A developmental intergroup perspective with adolescents”. For the purpose of this study, adolescents and their parents participated in two assessments (T1 and T2) with a time‐lag of 1 year between them. At T1, participating adolescents were 809 (49.81% female, 50.19% male; *M*_age_ = 15.61 years, *SD*_age_ = 1.12, range: 13.87–20.04 years). They were from two age groups: first‐ (*M*_age_ = 14.58 years, *SD*_age_ = 0.40, range 13.87–16.79) or third‐year (*M*_age_ = 16.64 years, *SD*_age_ = 0.47, range 15.84–20.04) students from secondary high schools in the Emilia‐Romagna region in the North‐East part of Italy. The majority of adolescents (86.88%, *M*_age_ = 15.62 years, *SD*_age_ = 1.11, range 13.87–20.04) were native Italians, while 13.12% (*M*_age_ = 15.55 years, *SD*_age_ = 1.18, range 13.88–18.29) had a migrant background (i.e., either they were born outside Italy or at least one of their parents was born outside Italy). Most of the families with a migrant background were from Eastern Europe, Africa, and Asia, though a small number of families came from America, Western Europe, and Oceania. In‐depth details about the countries of origin of families with a migrant background are reported in the Supplementary Materials (see Table S1). Furthermore, regarding the marital or relationship statuses of the families, most parents (75.03%) were married, while 14.73% were divorced, 8.12% cohabitating, 1.62% were widowed, and 0.50% were single.

In addition to adolescents, a total of 545 fathers (*M*_age_ = 51.19 years, *SD*_age_ = 5.03, range: 38–77 years) and 716 mothers (*M*_age_ = 48.11 years, *SD*_age_ = 4.68, range: 33–68 years) took part in the study. Regarding parents' educational level, most of them had a medium (i.e., high school diploma; 50.09% fathers, 51.85% mothers), followed by those with a high (i.e., university degree; 29.46% fathers, 36.04% mothers), and those with a low (i.e., elementary or middle school degree; 20.45% fathers, 12.11% mothers) educational level. Furthermore, fathers (30.36% left‐wing, 39.70% moderate, and 29.94% right‐wing) and mothers (37.02% left‐wing, 43.47% moderate, and 19.50% right‐wing) reported their political orientation.

All adolescents participated in the first assessment (T1), and 74.59% of them participated in T2. Regarding parents, they were included in the study if they participated at least in one of the two assessments (T1 or T2). The majority of them participated in both waves (53.58% fathers, 61.87% mothers), followed by those who took part only at T1 (41.28% fathers, 32.96% mothers) or at T2 (5.14% fathers, 5.17% mothers). As a preliminary check, we conducted missing value analyses for all participant samples (i.e., adolescents, fathers, and mothers). Little's (1988) Missing Completely at Random (MCAR) test yielded a normed χ^2^ (χ^2^/df = 2058.874/1802) of 1.14, indicating that data were likely missing completely at random. Therefore, all adolescents (*N =* 809) who joined the study at T1 and parents (545 fathers and 716 mothers) who took part at T1 or T2 were included in the analyses, and missing data were handled using the Full Information Maximum Likelihood (FIML) procedure in M*plus* 8.1 (Kelloway, 2015; Muthén & Muthén, 2017).

### Procedures

The study was approved by the Ethics Committee of Alma Mater Studiorum Univerisity of Bologna (Italy) as part of the ERC‐funded IDENTITIES “Managing identities in diverse societies: A developmental intergroup perspective with adolescents” project. This longitudinal research involved adolescents from several high schools in the Emilia‐Romagna region in the North‐East part of Italy, together with their parents. All the adolescents who participated in the study resided in Italy. Schools were selected through a stratified (by track and level of urbanization) randomized method, and principals were approached to present the project. Upon their approval, the study was then presented to students and their parents, who also received written and oral information about the study. Active consent from parents was obtained prior to their own and their children's participation. Active consent was also obtained from adolescents of age while their underage peers provided their assent to participate in the project. Participation in the study was voluntary, and adolescents and adults were informed that they could withdraw their consent at any time. The two data collections were conducted in January and February 2022 (T1) and January and February 2023 (T2).

### Measures

Adolescents and their parents completed a questionnaire including socio‐demographics (e.g., age, sex, and birth country) and a measure of attitudes toward migrant integration policies. In addition, adolescents reported their perception of discussion of current events with parents and the level of support they receive from them.

#### Attitudes toward migrant integration policies

Adolescents' and parents' attitudes toward migrant integration policies were assessed with the Attitudes toward Migrant Integration Policies scale (AMIP; Maratia et al., 2023). The instrument consists of eight items based on the Migrant Integration Policy Index (MIPEX). Participants received this prompt: “You will be presented with several policies for the integration of people with a migrant background. Please, rate how important it is that Italian national programs support policies to foster…” followed by one item for each policy area, as for example “…family reunion (e.g., accommodation, residence period)”. For each item, participants indicated their response on a 5‐point Likert scale (from 1 “Not at all important” to 5 “Absolutely important”). Cronbach's Alphas at T1 and T2 were 0.90 and 0.90 for adolescents, 0.91 and 0.93 for fathers, and 0.92 and 0.91 for mothers, respectively.

#### Discussion of current events with parents

The extent to which adolescents discuss current events with their parents was assessed at T1 with an ad hoc item [i.e., “Do you discuss current events (e.g., news, political events) within your family?”], which the adolescents rated on a 5‐point Likert scale (from 1 “Never” to 5 “Always”).

#### Perceived support from fathers and mothers

The extent to which adolescents feel supported by their fathers and mothers was assessed at T1 with a shortened version of the “Support” subscale of the Network Relationship Inventory (Furman & Buhrmester, 1985; for the Italian version, see Crocetti et al., 2011). Specifically, the shortened version of this scale included four items (e.g., “How much does your father/mother treat you like you're admired and respected?”), which the adolescents rated on a 5‐point Likert scale (from 1 “Not at all” to 5 “Very much”). At T1 Cronbach's Alphas were 0.88 and 0.87 for support perceived from fathers and mothers scales, respectively.

## RESULTS

### Preliminary analyses

Means, standard deviation, and within‐time correlations for all study variables are displayed in the Supplementary materials (see Table S2). As a preliminary step, we tested the measurement invariance of the Attitudes toward AMIP scale over time and across respondents. Results indicated that full metric invariance was reached (see Table S3). Therefore, we could proceed with the main analyses. Data, analysis codes, and outputs can be retrieved from the following OSF link: https://osf.io/by94k/.

### Cross‐lagged analyses

For the main purpose of the study, we conducted cross‐lagged panel analyses to model associations among adolescents', fathers', and mothers' attitudes as assessed with the AMIP scale (e.g., adolescents' AMIP at T1 predicting fathers' AMIP at T2, fathers' AMIP at T1 predicting adolescents' AMIP at T2), controlling for 1‐year (e.g., adolescents' AMIP at T1 predicting adolescents' AMIP at T2) stability paths, and within‐time correlations (e.g., correlation between adolescents' and fathers' AMIP at T1). The model was fully saturated. Furthermore, for each moderating variable, we conducted multigroup analyses to test the same model separately for each group, and the Wald test was used to perform pairwise comparisons.

#### Main results

**FIGURE 1:**
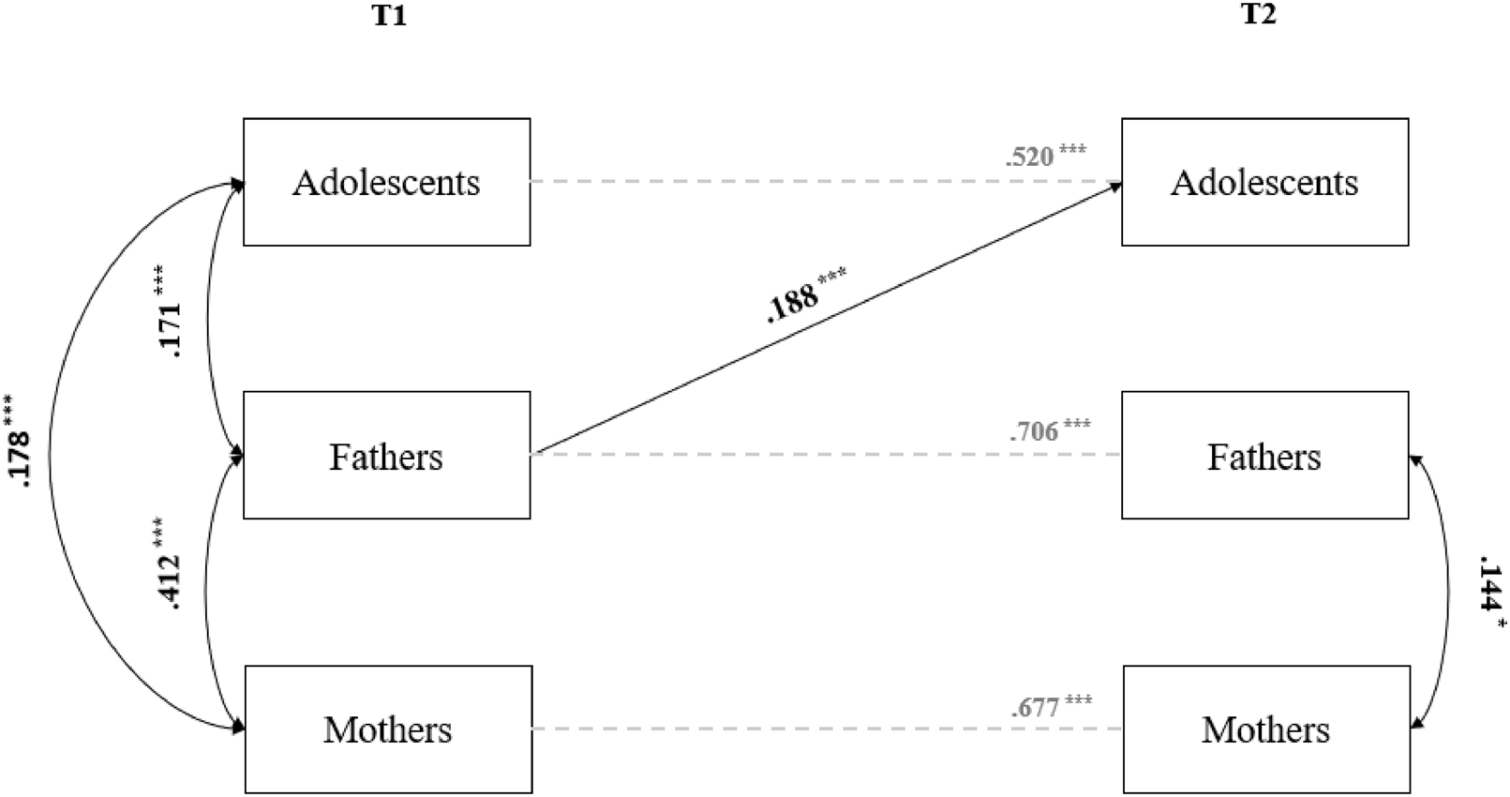
Results of the cross‐lagged panel model. *p < 0.05. ***p < 0.001.

Significant stability paths, cross‐lagged effects, and within‐time correlations are displayed in Figure 1. As can be seen from cross‐lagged paths, fathers, but not mothers, had a significant and positive effect on adolescents' AMIP over time, while adolescents' attitudes toward migrant integration policies did not predict parents' attitudes. Fathers (Wald test = 8.61, *p* = 0.003) and mothers (Wald = 4.485, *p* = 0.034) showed higher stability levels of their AMIP scores over time compared to their offspring, while no differences between fathers' and mothers' stability of their attitudes over time emerged. Finally, at T1, adolescents' scores correlated positively and significantly with both fathers' and mothers' scores, which were highly intertwined. At T2, only this latter association between fathers' and mothers' attitudes toward integration policies remained significant.

#### Multigroup analyses

We conducted multigroup tests to examine whether the results were moderated by adolescents' characteristics (i.e., sex, age), parents' characteristics (i.e., political orientation), and cultural (i.e., family ethnic background), and relational (i.e., discussion of current events, perceived support from fathers and mothers) family characteristics. Thus, we compared different groups of adolescents based on their sex (i.e., male vs. female) and age (i.e., first‐year vs. third‐year high‐school students). Furthermore, we identified different groups of parents based on their political orientation (i.e., left‐wing, moderate, and right‐wing). Moving to family‐related characteristics, we distinguished between families with (i.e., either adolescents were born outside Italy or at least one of their parents was born outside Italy) and without (i.e., adolescents and their parents were born in Italy) a migrant background. Finally, regarding family relational characteristics, for each moderator, we identified two different groups (e.g., low support vs. high support) of youth using the mean scores of adolescents on each variable at T1. Detailed results of the cross‐lagged effects and within‐time correlations linking adolescents' and parents' attitudes that differed significantly across groups, as indicated by the results of the Wald tests, are reported in Table 1.

**TABLE 1:** Standardized results of the cross‐lagged panel model with moderators.

	Cross‐lagged paths						Correlations			Correlated changes		
	T1 → T2						T1			T2		
Groups	A → F	A → M	F → A	M → A	F → M	M → F	A ↔ F	A ↔ M	M ↔ F	A ↔ F	A ↔ M	M ↔ F
**Total**	0.047	0.059	0.188^***^	0.063	0.059	0.028	0.171***	0.178***	0.412***	−0.035	0.020	0.144*
**Family members' characteristics**												
*Adolescents' sex*												
Male	0.123	0.006	0.260***	0.062	0.042	0.021	0.145*	0.157*	0.362***	−0.074	0.063	0.192*
Female	0.040	0.111*	0.131	0.068	0.086	0.019	0.198***	0.212***	0.458***	0.114	−0.060	0.134
*Adolescents' age*												
Younger	0.114	0.044	0.155**	0.128	0.047	0.044	0.163**	0.189**	0.449***	−0.117	0.100	0.183*
Older	−0.010	0.066	0.225***	0.010	0.059	−0.026	0.182**	0.168**	0.379***	0.092	0.054	0.130
*Fathers' political orientation*												
Left	**0.211**_**a**_ ^**^	−0.030	0.162	0.176	0.063	**−0.111**_**a**_	0.159	0.310***	0.474***	−0.144	**0.397**_**a**_ ^***^	**−0.026**_**a**_
Moderate	0.066_ab_	0.136	0.167**	0.021	0.134	−0.026_ab_	0.076	0.177*	0.378***	0.087	**−0.052**_**b**_	**0.381**_**b**_ ^**^
Right	−0.**091**_**b**_	0.171*	0.224**	0.134	−0.117	**0.109**_**b**_	0.240**	0.053	0.292***	−0.040	**−0.141**_**b**_	0.079_ab_
*Mothers' political orientation*												
Left	−0.004	0.102	0.155*	0.061	0.038	−0.004	0.179*	0.163**	0.383***	**0.242**_**a**_	**0.204**_**a**_ ^**^	0.114
Moderate	0.056	0.090	0.209**	0.069	0.098	0.005	0.090	0.097	0.346***	−0.166_ab_	**−0.063**_**b**_	0.113
Right	0.065	−0.030	0.325*	−0.086	−0.021	0.177	0.273**	0.168*	0.381**	**−0.167**_**b**_	**−0.140**_**b**_	0.190
**Family‐related characteristics**												
*Ethnic background*												
Italian	0.057	0.061	0.195***	0.058	0.037	0.009	0.175***	0.186***	0.421***	−0.069	0.019	0.130
Foreign	−0.184	0.022	0.102	0.095	0.281	0.190	0.162	0.140	0.298**	0.533	0.114	0.599**
*Discussion of current events*												
Low	0.009	0.089	0.224*	−0.026	0.100	**−0.112**	0.233***	0.210***	0.450***	−0.129	−0.092	0.161
High	0.096	0.057	0.182**	0.098	0.039	**0.128** ^*^	0.129*	0.138*	0.409***	0.041	0.061	0.047
*Support from father*												
Low	−0.021	0.128*	**−0.002**	0.173	0.019	**−0.162**	0.167*	0.107	0.507***	0.016	**−0.119**	0.142
High	0.078	0.020	**0.281** ^***^	0.036	0.064	**0.097**	0.174**	0.234***	0.367***	−0.052	**0.120**	0.179
*Support from mother*												
Low	−0.004	−0.009	0.205*	−0.023	0.023	−0.022	0.208**	0.142*	**0.510** ^***^	0.053	0.015	0.132
High	0.067	0.108*	0.174**	0.138**	0.079	0.081	0.145**	0.233***	**0.338** ^***^	0.188	0.046	0.131

*Note*: Bold font indicates significant differences across groups. In the case of more than two groups, subscript letters within columns indicate significant coefficient differences, as emerged from the Wald test.

Abbreviations: A, Adolescents; F, Fathers; M, Mothers.

* *p* < 0.05.

** *p* < 0.01.

*** *p* < 0.001.

##### Family members' characteristics

Regarding both adolescents' sex (i.e., male vs. female) and age (i.e., first‐year vs. third‐year high school students), the Wald tests showed no significant differences.

Regarding parents' characteristics, fathers ‘political orientation’ (i.e., left‐wing, moderate, right‐wing) moderated the cross‐lagged paths and the correlated changes of the association between adolescents' and parents' AMIP. Adolescents significantly and positively influenced fathers' attitudes over time when the latter reported having a left‐wing rather than a right‐wing political orientation (Wald test = 6.68, *p* = 0.009). At the same time, when fathers are left‐wing compared to when they are moderate (Wald test = 8.397, *p* = 0.003) or right‐wing (Wald test = 6.522, *p* = 0.010), the correlated changes between adolescents' and mothers' AMIP was stronger. Mothers' political orientation moderated only the correlated changes between parents' and adolescents' attitudes toward integration policies. Specifically, the association between mothers' and adolescents' AMIP at T2 was stronger when mothers reported being left‐wing than when they said being moderate (Wald test = 4.79, *p* = 0.028) or right‐wing (Wald test = 4.96, *p* = 0.025). Finally, when mothers are left‐wing rather than right‐wing (Wald test = 4.117, *p* = 0.042) politically oriented, the correlated change between adolescents' and fathers' inclusive attitudes was stronger.

##### Family‐related characteristics

Regarding family characteristics, family ethnic background (i.e., native Italian vs. families with a migrant background) did not moderate the transmission processes. Only a significant moderating effect of fathers' perceived support was found concerning family relational characteristics. Fathers' significant and positive influence on adolescents occurred only for youth who perceived high levels of support from fathers and not for those who perceived low support (Wald test = 7.63, *p* = 0.005).

## DISCUSSION

This study sought to disentangle intergenerational transmission processes by examining reciprocal associations among fathers', mothers', and adolescents' attitudes toward migrant integration policies. Furthermore, the role of potential factors, ranging from individual ones (e.g., adolescents' age group) to cultural (i.e., family ethnic background) and relational ones (e.g., fathers' support), that may either facilitate or hinder these processes were examined. The main study findings are discussed below in light of their implications.

### Intergenerational transmission of attitudes toward migrant integration policies

The primary purpose of this study was to unravel the family intergenerational transmission processes of attitudes toward migrant integration policies. In line with the hypothesis, this study found support for a unidirectional effect, with parents influencing adolescents' inclusive attitudes but not the other way around. This result is in line with studies suggesting that the impact of parents on their children is more substantial than vice versa (Vollebergh et al., 2001), especially when it comes to the study of attitudes. This predominance of parents' effects can be understood in light of the different degree of stability of attitudes toward integration policies shown by adults compared to adolescents, consistent with the principle that systems with a greater degree of stability are more likely to affect systems with a lower degree of stability (Asendorpf & Van Aken, 2003; Crocetti et al., 2016).

Furthermore, if baseline correlations are in line with cross‐sectional evidence suggesting that parents equally influence their children (e.g., Boonen, 2017), the current study found that changes in adolescents' inclusive attitudes over time were associated only with fathers', but not with mothers' inclusive attitudes. This result may be attributed to the specific attitudes considered in this study and their relation with politics and policymakers. Regarding political participation, a consistent gender gap persists within families, where fathers generally exhibit greater political interest (Burns et al., 2001) and engagement (Boonen, 2017) than mothers. Furthermore, fathers tend to spend more time with their children than fathers from previous generations (McGill, 2014) and show increased family involvement and emotional closeness to their children (Petts et al., 2018). In line with this reasoning, fathers can exert a stronger impact on the development of political attitudes of their offspring, including the attitudes toward integration policies, as shown by the present research.

### The moderators of the parental transmission

The second aim of this study was to understand which individual characteristics of both adolescents (e.g., adolescent age) and their parents (e.g., political orientation) can facilitate the intergenerational transmission of inclusive attitudes. Furthermore, cultural (i.e., family ethnic background) and relational (e.g., perceived support from fathers) family‐related characteristics were analyzed to understand which factors can moderate parents' influence on their children. The results showed that fathers' individual characteristics and family relational factors can enhance intergenerational transmission processes.

#### Individual characteristics of family members

Adolescents, their fathers, and their mothers are part of a complex system in which each family member has a high level of interdependence with the others (Scott et al., 2018). Thus, the individual characteristics of a specific member can play a crucial role in influencing the development of other family members over time. Concerning adolescents' characteristics, however, the present study found no effect of sex (i.e., male vs. female). This result does not confirm the presence of a gender‐match effect according to which attitudes' similarities are more substantial in same‐gender dyads (e.g., mother–daughter; Filler & Jennings, 2015). Instead, they highlighted that the association between parents' and children's inclusive attitudes is the same despite the sex of the adolescents, as suggested by previous evidence (Augustijn, 2022; Bucx et al., 2010; Degner & Dalege, 2013; Duriez et al., 2008; Meeusen & Dhont, 2015).

Similarly to what emerged with sex, adolescents' age groups (i.e., first‐year vs. third‐year high school students) did not moderate the family transmission of attitudes toward migrant integration policies, in contrast with results suggesting a reduction of family's influence as adolescents grow up and start to interact with significant others (Alfieri & Marta, 2012; De Goede et al., 2009). Conversely, this result aligns with other evidence suggesting that parental influence regarding specific attitudes or values continues to be consistent throughout and can extend beyond adolescence. In particular, considering socio‐political attitudes (e.g., political orientation), similarities between parents and their offspring persist as the latter have reached adulthood (Willoughby et al., 2021). In light of this, adolescents' and parents' attitudes toward integration policies, given their connection to the political context, may therefore show the same trend.

Moving to parents' characteristics, a nuanced pattern of findings emerged when considering their political orientation. First, this study found that when fathers are left‐wing‐oriented, the one‐way transmission becomes bi‐directional, with adolescents positively influencing their fathers' inclusive attitudes. This result suggests greater openness of left‐wing fathers toward their children's views compared to fathers who are right‐wing oriented. One possible explanation could be that individuals with right‐wing preferences often exhibit more conservative and authoritarian attitudes (see Van Hiel et al., 2010) and a general resistance to change (Jost et al., 2003). This evidence is of particular interest, as it shows that, on the one hand, left‐wing parents show more openness toward their children's perspectives. On the other hand, fathers' influence on adolescents' inclusive attitudes was the same despite the political orientation of the former.

Second, the findings highlighted that parents' political orientation moderated the correlated changes. Specifically, when both parents reported being left‐wing, the correlations between adolescents' and mothers' attitudes became more robust over time. In addition, when mothers reported to be left‐wing, the correlation between adolescents' and fathers' attitudes became stronger. Overall, these results highlight that adolescents' and parents' inclusive attitudes become intertwined over time, especially in left‐wing families in which views of themes related to immigration can represent a pivot of political attitudes (Rekker, 2016).

In light of these results, more studies are needed to understand the role of fathers' and mothers' political orientation in the transmission of positive intergroup attitudes and which are the factors (e.g., openness to change) that could make the transmission processes not a one‐way transfer from parents to children but a two‐way process where the latter also can make the difference. In this vein, the results proved to be quite interesting, suggesting that, under specific conditions (i.e., left‐wing fathers), adolescents may also have an active role in the intergenerational transmission processes, as hypothesized by advancements of intergenerational transmission theories and research (e.g., Akyil et al., 2016; Barni et al., 2023; De Mol et al., 2013; Kuczynski et al., 2016; Miklikowska, 2016).

#### Is it a matter of culture or relationships? The importance of feeling supported

The individual characteristics of fathers, mothers, and their children alone may not be sufficient to disentangle the intergenerational transmission processes, a complex and dynamic phenomenon such as the context in which it is embedded. Indeed, it may be necessary to consider other aspects related to the family system, such as its culture (i.e., ethnic background) and relationships (i.e., family discussion of current events and perceived support from parents). In this study, a predominance of relational factors was documented.

Although this study hypothesized a moderating effect of family ethnic background (i.e., native Italian vs. families with a migrant background), the results did not support the expectations. Fathers' positive influence on adolescents' inclusive attitudes was the same whether they have a migrant background or not. These results align with evidence suggesting that family relational characteristics may have a crucial role (Brown et al., 2018; Miklikowska, 2016) in moderating the transmission processes.

To unravel this possible influence of family relational factors, this research focused on two facets by examining whether the frequency with which adolescents discuss current events with their parents and the perceived support they receive from the latter might exert an effect on the transmission of positive attitudes toward integration policies. On the one hand, this study did not find any moderating effect of family discussions, in contrast to the hypothesis that increased frequency of discussion would lead to more opportunities for socialization and thus heightened parental transmission (e.g., Meeusen & Boonen, 2022). On the other hand, results indicated that the quality of these relationships, as indicated by the support perceived by adolescents, moderates the transmission from father to child and the correlated changes between mothers' and adolescents' inclusive attitudes. This evidence aligns with prior research in which adolescents reporting greater parental support exhibited attitudes (i.e., prejudices) more in line with those of their parents (Miklikowska, 2016). In addition to corroborating these earlier findings, this result again underscores the paternal figure's key role in transmitting these attitudes. Indeed, only the support perceived by adolescents from their fathers moderates the transmission processes of attitudes toward integration policies. Specifically, the more adolescents report receiving support from their fathers, the stronger the positive influence exerted by the latter on their children's inclusive attitudes. Thus, through their supportiveness, fathers establish a relational foundation that can foster alignment with their children's views, as previous research has already suggested (Augustijn, 2022; Brown et al., 2018; Miklikowska, 2016).

Taken together, these results highlight that family relational factors are more relevant than the family's cultural aspects in moderating the influence of fathers on their children's attitudes toward migrant integration policies. Furthermore, the quality of these relationships, specifically the extent to which adolescents feel supported by their fathers, rather than their quantity, in terms of frequency of discussions, plays a crucial role in the intergenerational transmission processes. Thus, emotional warmth and supportiveness within family relationships profoundly impact adolescents' inclusiveness and their propensity to embrace fathers' attitudes toward integration policies, demonstrating the multifaceted nature of familial influence over time.

### Theoretical and practical implications

This study advances the theoretical understanding of the family intergenerational transmission processes and their individual or family‐contextual moderators. In this vein, analyzing the impact of each family member's characteristics over time provides a greater understanding of the potential reciprocal nature of this phenomenon (Acock & Bengtson, 1980) and allows for more robust associations (Branje et al., 2020). The results have indeed indicated that, when considering attitudes toward migrant integration policies, the transmission is primarily a unidirectional phenomenon, with fathers but not mothers influencing their children's attitudes. Nevertheless, when considering specific parents' characteristics (i.e., political orientation), the transmission of attitudes can become bidirectional, with adolescents influencing their fathers. Furthermore, by focusing on the cultural and relational characteristics of the family, this study highlights the conditions under which it is possible to enhance the transmission processes. Specifically, it allows for a deeper exploration of the effect of adolescent‐perceived support from parents, and in particular fathers, on family transmission, a facet that has been studied in a few previous studies (Augustijn, 2022; Brown et al., 2018; Miklikowska, 2016).

Together with theoretical insights, the current study has important practical implications. The results have shown how the likelihood of transmitting inclusive attitudes from generation to generation is greatly influenced by the quality of family relationships, particularly the support perceived by fathers. Therefore, to successfully promote parent–child value transmission (Augustijn, 2022; Brown et al., 2018; Miklikowska, 2016), therapists working with families might explore how parents communicate with their children, emphasizing the importance of warm relationships and language while taking into account the different meanings of closeness depending on the family's culture (Akyil et al., 2016). Furthermore, this study has demonstrated how transmission can sometimes be bidirectional, meaning that parents, specifically left‐wing political‐oriented fathers, are also open to their children's values and attitudes. This finding could help to understand what promotes the negotiation of different values between parents and children rather than a polarization of their ideals and attitudes, which often leads to family conflicts (Iyengar et al., 2018). In this sense, therapy could be a valuable space for parents and children to analyze the similarities and differences in their values (Akyil et al., 2016), thus discussing how different political orientations may affect their relationships (Chan et al., 2018) and, thus, the values and attitudes transmission.

### Limitations and future directions

Overall, this study should be considered in light of some shortcomings that can suggest venues for future research. In this vein, a longitudinal study with two annual waves may not fully disentangle the complexity of the family transmission processes. Indeed, although this study focused on two cohorts of students from different age groups, following adolescents for the entire high school period would make it possible to understand better how parental influence changes over time. Furthermore, future studies could further enrich available knowledge by considering the transition from adolescence to young adulthood, which still represents a critical life stage for developing attitudes (e.g., Bobba et al., 2023; Rekker, 2016).

Second, the more significant influence exerted by fathers rather than mothers on adolescents' attitudes toward integration policies could be explained by the fact that the attitudes analyzed in this study are closely tied to politics. Numerous studies have shown the continued presence of a gender gap (Boonen, 2017; Burns et al., 2001; Marien et al., 2010) in family dynamics regarding political engagement. For this reason, future studies should investigate whether the current results are replicated when considering different intergroup attitudes, such as prejudices.

Finally, it should be noted that specific characteristics of the context of this study, such as ethnic diversity concentration and the political scenario, may influence the study results. Italy has been considered one of the major immigrant destination countries in Europe (United Nations, 2019), and Emilia‐Romagna, the specific context of the current study, is a region characterized by unique features in terms of the concentration of people with a migrant background (Regione Emilia‐Romagna, 2023). Furthermore, during the first 2 years of the study, the Italian political scenario changed, moving from a left‐wing government to a right‐wing one. The higher concentration of individuals with a migrant background (Kunst et al., 2021), on the one hand, and the different approaches to integration policies between these two political alignments (Geddes & Pettrachin, 2020), on the other hand, may have played a role in influencing the salience of immigrants‐related attitudes (Bobba, Thijs, & Crocetti, 2024) and their transmission. Future studies should also examine how varying ethnic compositions and political landscapes might affect the transmission of attitudes toward integration policies and whether this study's findings apply to other contexts.

## CONCLUSION

This longitudinal study involving three family members (adolescents, their fathers, and mothers) demonstrates the intergenerational transmission of attitudes toward migrant integration policies, with fathers having a stronger positive effect than mothers on adolescents' inclusive attitudes. Furthermore, when considering the moderating effect of specific individual factors (i.e., fathers' political orientation), this study found that this transmission could become bi‐directional, with adolescents also influencing their fathers' attitudes toward migrant integration policies. Finally, the more the adolescents reported a high level of fathers' support, the more their attitudes align with the latter's. Thus, the current study underscores the complexity of the family context, highlighting that the transmission of inclusive attitudes does not always operate in one way or another but, in some cases, both simultaneously.

## FUNDING INFORMATION

This work was supported by a grant from the European Research Council (ERC) under the European Union's Horizon 2020 research and innovation programme (ERC‐CoG IDENTITIES Grant agreement No. [101002163]; Principal investigator: Elisabetta Crocetti). Open access publishing facilitated by Universita degli Studi di Bologna, as part of the Wiley ‐ CRUI‐CARE agreement [Correction added on 17 July 2024, after first online publication: CRUI‐CARE funding statement has been added.]

## ETHICS STATEMENT

All procedures performed in this study involving human participants were in accordance with the ethical standards of the Ethics Committee of the Alma Mater Studiorum University of Bologna (Italy) and with the 1964 Helsinki declaration and its later amendments or comparable ethical standards.

## Supporting information


Data S1

## Data Availability

Data, analysis codes, and outputs are available at the following OSF link: https://osf.io/by94k/.
