# Supplementary material for: One way or another…or both: Different roles of fathers, mothers, and adolescents in the intergenerational transmission of inclusive attitudes
Source: Fam Process. 2024 Jun 24;63(4):2505–26. doi: 10.1111/famp.13023 (PMC11659112; doi:10.1111/famp.13023)
Supplement: Supplementary file 1 — Data S1 [file FAMP-63-2505-s001.docx]

**Supplemental Materials for**

**“One Way or Another…or Both: Different Roles of Fathers, Mothers, and Adolescents in the Intergenerational Transmission of Inclusive Attitudes”**

**Table S1.**

Country of origin of family with a migrant background.

|  | Fathers’ country of origin | |  | Mothers’ country of origin | |  | Adolescents’ country of origin | |
| --- | --- | --- | --- | --- | --- | --- | --- | --- |
|  | *n* | % |  | *n* | % |  | *n* | % |
| Albania | 3 | 12.49 |  | 9 | 13.83 |  | 3 | 11.52 |
| Argentina | 1 | 4.17 |  | 1 | 1.54 |  | - | - |
| Australia | - | - |  | 1 | 1.54 |  | 1 | 3.85 |
| Bolivia | - | - |  | - | - |  | 1 | 3.85 |
| Brazil | - | - |  | 1 | 1.54 |  | - | - |
| Cambodia | - | - |  | - | - |  | 1 | 3.85 |
| Canada | 1 | 4.17 |  | - | - |  | - | - |
| China | 3 | 12.49 |  | 4 | 6.14 |  | 1 | 3.85 |
| Ivory Coast | - | - |  | 2 | 3.08 |  | - | - |
| Croatia | - | - |  | 1 | 1.54 |  | - | - |
| Denmark | - | - |  | 1 | 1.54 |  | - | - |
| Egypt | - | - |  | 1 | 1.54 |  | 1 | 3.85 |
| Ethiopia | - | - |  | - | - |  | 1 | 3.85 |
| France | - | - |  | 1 | 1.54 |  | - | - |
| Germany | 1 | 4.17 |  | 4 | 6.14 |  | - | - |
| Ghana | - | - |  | 1 | 1.54 |  | 1 | 3.85 |
| India | 1 | 4.17 |  | 1 | 1.54 |  | 2 | 7.68 |
| Israel | 1 | 4.17 |  | - | - |  | - | - |
| North Macedonia | 1 | 4.17 |  | 1 | 1.54 |  | - | - |
| Morocco | 1 | 4.17 |  | - | - |  | - | - |
| Mauritius | - | - |  | 1 | 1.54 |  | - | - |
| Nepal | - | - |  | - | - |  | 1 | 3.85 |
| Nigeria | 2 | 8.32 |  | 3 | 4.62 |  | 3 | 11.52 |
| Netherlands | 1 | 4.17 |  | - | - |  | 1 | 3.85 |
| Paraguay | - | - |  | 1 | 1.54 |  | - | - |
| Poland | - | - |  | 3 | 4.62 |  | - | - |
| Czech Republic | - | - |  | 1 | 1.54 |  | - | - |
| Republic of Moldova | - | - |  | 3 | 4.62 |  | 1 | 3.85 |
| Dominican Republic | 1 | 4.17 |  | 1 | 1.54 |  | 1 | 3.85 |
| Romania | 5 | 20.83 |  | 7 | 10.75 |  | 2 | 7.68 |
| Russia | - | - |  | 2 | 3.08 |  | - | - |
| Serbia | - | - |  | 2 | 3.08 |  | - | - |
| Slovakia | - | - |  | 2 | 3.08 |  | - | - |
| Spain | - | - |  | 1 | 1.54 |  | 1 | 3.85 |
| South Africa | - | - |  | 1 | 1.54 |  | 1 | 3.85 |
| Sweden | - | - |  | 1 | 1.54 |  | - | - |
| Switzerland | 1 | 4.17 |  | 2 | 3.08 |  | - | - |
| Togo | - | - |  | 1 | 1.54 |  | 1 | 3.85 |
| Tunisia | 1 | 4.17 |  | 3 | 4.62 |  | - | - |
| USA | - | - |  | 1 | 1.54 |  | 1 | 3.85 |
| Vietnam | - | - |  | - | - |  | 1 | 3.85 |
|  |  |  |  |  |  |  |  |  |
| Total | 24 | 100% |  | 65 | 100% |  | 26 | 100% |

*Note*. *n=*Number of participants; %= Percentage of participants*.*

**Table S2**

Means, standard deviations, and correlations among study variables.

|  | *M* | *SD* | 1. | 2. | 3. | 4. | 5. | 6. | 7. | 8. | 9. | 10. | 11. | 12. | 13. | 14. | 15. | 16. |
| --- | --- | --- | --- | --- | --- | --- | --- | --- | --- | --- | --- | --- | --- | --- | --- | --- | --- | --- |
| 1. ASex |  |  | - |  |  |  |  |  |  |  |  |  |  |  |  |  |  |  |
| 2. AgeG |  |  | .03 | - |  |  |  |  |  |  |  |  |  |  |  |  |  |  |
| 3. Fed |  |  | .01 | -.03 | - |  |  |  |  |  |  |  |  |  |  |  |  |  |
| 4. Fpo |  |  | -.02 | .09 | -.16^**^ | - |  |  |  |  |  |  |  |  |  |  |  |  |
| 5. Med |  |  | -.07 | .02 | .43^**^ | -.10 | - |  |  |  |  |  |  |  |  |  |  |  |
| 6. Mpo |  |  | -.12^**^ | .05 | -.06 | .40^***^ | -.16^***^ | - |  |  |  |  |  |  |  |  |  |  |
| 7. Abackgr |  |  | .06 | -.08^*^ | .03 | .04 | -.05 | .09^*^ | - |  |  |  |  |  |  |  |  |  |
| 8. Fdisc T1 | 3.60 | 0.98 | .02 | .04 | .08 | -.01 | .14^***^ | -.04 | -.12^***^ | - |  |  |  |  |  |  |  |  |
| 9. Suppf T1 | 3.84 | 0.84 | -.07 | .01 | .04 | -.01 | .09^*^ | -.03 | -.12^***^ | .14^***^ | - |  |  |  |  |  |  |  |
| 10. Suppm T1 | 4.08 | 0.75 | -.07 | .01 | .01 | .00 | .10^*^ | -.01 | -.08^*^ | .13^***^ | .51^***^ | - |  |  |  |  |  |  |
| 11. AAMIP T1 | 4.05 | 0.71 | .18^***^ | -.04 | .13^**^ | -.13^**^ | .10^**^ | -.23^***^ | .03 | .09^**^ | .21^***^ | 15^***^ | - |  |  |  |  |  |
| 12. FAMIP T1 | 3.72 | 0.79 | .03 | -.03 | .28^***^ | -.37^***^ | .20^***^ | -.27^***^ | .08 | .06 | .027 | .02 | .20^***^ | - |  |  |  |  |
| 13. MAMIP T1 | 3.82 | 0.69 | .00 | -.08^*^ | .21^***^ | -.28^***^ | .25^***^ | -.39^***^ | .09^*^ | .13^**^ | -.01 | -.02 | .20^***^ | .42^***^ | - |  |  |  |
| 14. AAMIP T2 | 3.87 | 0.71 | .20^***^ | .00 | .22^***^ | -.21^***^ | .18^***^ | -.28^***^ | .04 | .10^*^ | .155^***^ | .15^***^ | .56^***^ | .36^***^ | .27^***^ | - |  |  |
| 15. FAMIP T2 | 3.63 | 0.81 | -.09 | -.04 | .30^***^ | -.34^***^ | .15^*^ | -.24^***^ | .01 | .12^*^ | .11 | .11 | .20^***^ | .72^***^ | .38^***^ | .30^***^ | - |  |
| 16. MAMIP T2 | 3.76 | 0.69 | .06 | -.10^*^ | .27^***^ | -.32^***^ | .28^***^ | -.37^***^ | .05 | .10^*^ | .02 | .03 | .22^***^ | .36^***^ | .71^***^ | .23^***^ | .36^***^ | - |

*Note*. ASex= Adolescents’ sex; ASex: 0=male, 1=female. AgeG= Adolescents’ age group; AgeG: 0=first year, 1=third year. Fed= Father educational level; Med= Mother educational level; Fed/Med: 0=none, 1=high school, 2=master degree. Fpo= Father political orientation; Mpo= Mother political orientation; Fpo/Mpo: from 0 (extreme left) to 10 (extreme right). Abackgr: adolescents’ ethnic background; Abackgr: 0=Italian, 1=Foreign. Fdisc= Family discussion of actuality; Suppf/Suppm= adolescents perceived support from father/mother; AAMIP= Adolescents’ Attitudes toward Migrant Integration Policies; FAMIP= Fathers’ Attitudes toward Migrant Integration Policies; MAMIP= Mothers’ Attitudes toward Migrant Integration Policies.

^*^ *p* < .05; ^**^ *p* < .01; ^***^ *p* < .001

**Longitudinal and Multigroup Measurement Invariance of Study Variables**

As a preliminary step, the measurement invariance of the Attitudes toward Migrant Integration Policies scale (AMIP) was tested over time and across respondents. First, longitudinal measurement invariance of the AMIP scale for adolescents, fathers, and mothers was tested. Next, multigroup measurement invariance of AMIP scale was tested across the adolescents’, fathers’, and mothers’ samples.

For both longitudinal and multigroup invariance, configural and metric levels were tested, since metric invariance is the minimum requirement for cross-lagged panel analyses. To this end, the configural models function as baseline models and should therefore display a good fit, evaluated based on the following criteria: the Comparative Fit Index (CFI) with values higher than .90 and .95 indicative of an acceptable and very good fit; the Root Mean Square Error of Approximation (RMSEA) and the Standardized Root Mean Residual (SRMR) with values below .08 and .05 indicative of an acceptable and very good fit (Byrne, 2012); and the RMSEA’s 90% confidence interval’s upper bound lower than .10 indicating an acceptable fit of the model (Chen et al., 2008). In order to establish metric invariance (i.e., constraining factor loadings to be equal across time and/or groups), changes in fit indices from the configural to the metric model were evaluated (e.g., Cheung & Rensvold, 2002). Specifically, a significant Δχ_SB_^2^ (Satorra & Bentler, 2001), and ΔCFI ≥ -.010 supplemented by ΔRMSEA ≥ .015 (Chen, 2007) indicates non-invariance. Results are displayed in Table S3. As can be inferred, full metric invariance was reached in all analyses.

**Table S3**

Longitudinal and multigroup measurement invariance of the AMIP scale

| Models |  | | | | |  | Model comparisons | | | |
| --- | --- | --- | --- | --- | --- | --- | --- | --- | --- | --- |
|  | χ^2^ | df | CFI | SRMR | RMSEA [90% CI] |  | Models | Δχ_SB_^2^ | ΔCFI | ΔRMSEA |
| Longitudinal Invariance | | | | | | | | | | |
| AMIP (Adolescents) | | | | | | | | | | |
| Configural (M1) | 422.607 | 95 | .923 | .043 | .067 [.060, .073] |  |  |  |  |  |
| Metric (M2) | 443.044 | 102 | .920 | .052 | .066 [.059, .072] |  | M2-M1 | 15.563 (7) | -.003 | -.001 |
| AMIP (Fathers) | | | | | | | | | | |
| Configural (M1) | 310.757 | 95 | .928 | .044 | .066 [.058, .074] |  |  |  |  |  |
| Metric (M2) | 341.835 | 102 | .920 | .071 | .067 [.060, .075] |  | M2-M1 | 32.809 (7) | -.008 | .001 |
| AMIP (Mothers) | | | | | | | | | | |
| Configural (M1) | 395.604 | 95 | .925 | .047 | .68 [.061, .75] |  |  |  |  |  |
| Metric (M2) | 404.704 | 102 | .925 | .051 | .066 [.059, .073] |  | M2-M1 | 5.490 (7) | .000 | -.002 |
| Multigroup Longitudinal Invariance | | | | | | | | | | |
| AMIP | | | | | | | | | | |
| Configural (M1) | 2365.573 | 1041 | .912 | .053 | .040 [.038, .042] |  |  |  |  |  |
| Metric (M2) | 2506.296 | 1076 | .905 | .065 | .041 [.039, .043] |  | M2-M1 | 139.775 (35) | -.007 | .001 |

*Note*. M = model; χ^2^ = chi-square; df = degree of freedom; CFI = Comparative Fit Index; SRMR = Standardized Root Mean Square Residual;

RMSEA = Root Mean Square Error of Approximation; CI = confidence interval; Δ = change in the parameter.

^*^ *p* < .05; ^***^ *p* < .001
